# Idiosyncrasy and generalizability of contraceptive- and hormone-related functional connectomes across the menstrual cycle

**DOI:** 10.1162/netn.a.20

**Published:** 2025-07-29

**Authors:** Katherine L. Bottenhorn, Taylor Salo, Emily G. Jacobs, Laura Pritschet, Caitlin Taylor, Megan M. Herting, Angela R. Laird

**Affiliations:** Department of Population and Public Health Sciences, University of Southern California, Los Angeles, CA, USA; Department of Psychology, Florida International University, Miami, FL, USA; Lifespan Informatics and Neuroimaging Center (PennLINC), Department of Psychiatry, Perelman School of Medicine, University of Pennsylvania, Philadelphia, PA, USA; Lifespan Brain Institute (LiBI) of Penn Medicine and CHOP, Philadelphia, PA, USA; Neuroscience Research Institute, University of California, Santa Barbara, CA, USA; Department of Psychological and Brain Sciences, University of California, Santa Barbara, CA, USA; Department of Physics, Florida International University, Miami, FL, USA; Center for Imaging Science, Florida International University, Miami, FL, USA

**Keywords:** Functional connectivity, Estradiol, Progesterone, Hormonal contraceptives, Menstrual cycle

## Abstract

Neuroendocrinology has received little attention in human neuroscience research, resulting in a dearth of knowledge surrounding potent and dynamic modulators of cognition and behavior, as well as brain structure and function. This work addresses one such phenomenon by studying functional connectomics related to ovarian hormone fluctuations throughout the adult menstrual cycle. To do so, we used fMRI and hormone assessments from two dense, longitudinal datasets to assess variations in functional connectivity with respect to endogenous and exogenous endocrine factors throughout the menstrual cycle. First, we replicated prior findings that common, group-level, and individual-specific factors have similar relative contributions to functional brain network organization. Second, we found widespread connectivity related to hormonal contraceptive (HC) use, in addition to sparser estradiol- and progesterone-related connectivity. Differential generalizability of these connectivity patterns suggests progestin-specific impacts on functional brain organization in HC users. These results provide novel insight into within-individual changes in brain organization across the menstrual cycle and the extent to which these changes are shared between individuals, illuminating understudied phenomena in reproductive health and important information for all neuroimaging studies that include participants who menstruate.

## Author Summary

Endocrine modulation of brain function across the menstrual cycle is poorly understood. Human neuroimaging research on the menstrual cycle has long relied on group differences and/or coarse, within-individual cycle stage differences, overlooking considerable individual differences in brain organization, the menstrual cycle, and hormone concentrations. Here, we take a multidataset approach to identify the idiosyncratic contraceptive- and hormone-related functional connectivity from within-individual neuroendocrine dynamics and then test the generalizability of this connectivity to other individuals. In doing so, we identified idiosyncratic hormone-responsive functional connectivity that is somewhat generalizable to other individuals, although this generalizability is complicated by hormonal contraceptive use, potentially reflecting differential connectivity between contraceptive formulations. Thus, this work illuminates individual similarities and differences in neuroendocrine dynamics across the menstrual cycle.

## INTRODUCTION

As a critical node of the endocrine system, the brain influences and is influenced by peripheral hormone fluctuations, which can impact its structure and function (Albert et al., 2017; Beltz & Moser, 2020; Brown et al., 2015; Galea et al., 2017; Taylor et al., 2020). A prime example of this bidirectional relationship comes from fluctuating ovarian hormones (e.g., estradiol and progesterone) across the menstrual cycle, which are integral to the female reproductive system. Estradiol influences dendritic spine density (Hao et al., 2006), major neurotransmitter systems (Barth et al., 2015; Bendis et al., 2024; Diekhof, 2018; Zsido et al., 2017), learning and memory (Jacobs & D’Esposito, 2011; Jacobs et al., 2016; Taxier et al., 2020), and emotion regulation (Graham et al., 2017; van Wingen et al., 2011). Conversely, progesterone’s role in cognition is largely inconclusive (Henderson, 2018), although it is related to significant fluctuations in medial temporal lobe volume (Taylor et al., 2020; Zsido et al., 2023). Hormonal contraceptives (HCs) with synthetic estrogens and progestins disrupt this cycle. Furthermore, HC use has been linked to increased risk of depression (Skovlund et al., 2016); altered gray matter (GM) volume in medial temporal, prefrontal cortex (PFC), and hypothalamic regions (Brønnick et al., 2020; Chen et al., 2021); and differences in cognitive function (Brønnick et al., 2020; Warren et al., 2014).

However, endocrine factors have received little attention in human neuroscience in either sex (Taylor et al., 2021; Will et al., 2017). Of the more than 50,000 published human MRI studies, only 0.5% address sex hormones or other endocrine factors (Jacobs, 2023b; Taylor et al., 2021). Here, we focus on ovarian hormone fluctuations during the adult menstrual cycle: a period of 25–30 days, during which estradiol levels undergo ~eightfold changes and progesterone levels undergo ~80-fold changes. Open questions remain regarding the roles of these daily and weekly hormone fluctuations in brain function, in addition to the influences of HC use (Pletzer & Kerschbaum, 2014). Much of the extant literature focuses on contrasts between menstrual cycle phases, which obfuscate hormone dynamics, as endogenous estradiol and progesterone fluctuations are not characterized by discrete transitions between menstrual cycle phases but curvilinear progressions across the cycle. Within the remarkably undersized proportion of neuroimaging papers that address endocrine factors, fewer than 8% of published human neuroimaging studies of the menstrual cycle have assessed individuals at more than three time points and only 30% assessed individuals more than twice (Dubol et al., 2021). Furthermore, menstrual cycles vary within an individual as much as they do between individuals (Fehring et al., 2006; Jasienska & Jasienski, 2008; Stern et al., 2022). Studies of exogenous hormone manipulations, on the other hand, suggest that HC use creates a hyperprogestogenic state in the brain characterized by altered medial temporal, prefrontal, and parieto-occipital connectivity (reviewed by Casto et al., 2022). However, extant studies of both endogenous and exogenous hormones across the menstrual cycle focus largely on between-group differences and are unable to model or estimate individual differences, or idiosyncrasies, in these effects. Thus, the attention paid to ovarian hormones and endocrine rhythms in the extant human neuroimaging literature is insufficient to characterize neuroendocrine dynamics across the menstrual cycle. Given the expression of ovarian hormone receptors across the brain *and* the interactions of ovarian hormone metabolites with major neurotransmitter systems (Barth et al., 2015; Baulieu et al., 1996; Brinton et al., 2008), these fluctuations have large, currently overlooked consequences for brain function.

Deep phenotyping and precision neuroscience studies can address this gap by densely sampling individuals with repeated measurements over days, weeks, and months to better understand both between- and within-individual variability in the brain (Gordon et al., 2017; Jacobs, 2023a; Poldrack, 2017; Poldrack et al., 2015; Pritschet et al., 2021; Pritschet & Jacobs, 2024). Deep and/or dense phenotyping approaches allow for more precise measurement and estimation of previously unmodeled sources of individual differences. Gratton et al. (2018) found that common group factors and stable individual-specific factors have near-equal contributions to similarity of functional brain networks, illuminating the balance of generalizability and idiosyncrasy in functional connectomics. While the same work found that within-individual differences over time are responsible for a relatively small proportion of network similarity, deep phenotyping of the human brain across the menstrual cycle shows notable changes in the medial temporal lobe structure, in addition to cortical and cerebellar functional network organization (Fitzgerald et al., 2020; Pritschet et al., 2020; Taylor et al., 2020). While dense sampling approaches offer a nearly unparalleled opportunity to better elucidate hormone–brain interactions, only a minority of such studies include endocrine assessments (Arélin et al., 2015; Barth et al., 2016; Filevich et al., 2017; Pritschet et al., 2020; Zsido et al., 2023). Here, we use three such datasets to investigate the roles of ovarian hormones in brain network organization across the menstrual cycle. The first two are from the 28andMe projects, which include daily fMRI, hormone, and behavioral assessments over two 30-day periods in a single premenopausal woman while naturally cycling (NC; 28andMe; Pritschet et al., 2020) and again using HCs (28andOC) after a 10-month wash-in period. The third is from the Dense Investigation of Variability in Affect (DIVA) Study (Bottenhorn et al., 2024), which includes weekly fMRI, hormone, and behavioral assessments over 2–5 weeks in three premenopausal women: one NC and two using HCs. These data present an opportunity to study variability in the brain related to the menstrual cycle and to better understand neuroendocrine dynamics affecting over 50% of the population.

The present study examines the roles of HC use and ovarian hormone concentrations in functional brain network organization across the menstrual cycle. First, we aimed to replicate the findings of Gratton et al. (2018) regarding the degrees of generalizability and idiosyncrasy in functional network organization within our multidataset sample and extend this approach to assess network similarity between different datasets and HC use. To do this, we calculated the similarity (i.e., product–moment correlation) between functional brain networks across each category (i.e., group, dataset, HC use, individual). Second, we identified the roles of HC use and ovarian hormones in the brain’s functional connectome by a combination of graph theory and machine learning. To this end, we used the network-based statistic in a machine learning framework (NBS-Predict; Serin et al., 2021; Zalesky et al., 2010) to identify HC- and hormone-sensitive functional connectivity in the 28andMe and 28andOC datasets. We then assessed how such connectivity generalized to the independent DIVA dataset. This multivariate, predictive connectomics approach is more generalizable and robust than univariate, descriptive approaches (Mantwill et al., 2022; Marek et al., 2022; Shen et al., 2017). We expected that dataset- and individual-specific features would dominate functional connectivity, compared with HC use, and that brain regions demonstrating high concentrations of estrogen receptors (e.g., PFC, hippocampus, and broader medial temporal lobe; Wang et al., 2010) would be involved in hormone-sensitive connectivity over the course of the menstrual cycle. Investigating the interplay of endocrine factors in functional connectomics on daily and weekly scales will improve our understanding of the brain, contribute to a growing literature regarding neuroendocrine dynamics in both sexes (Ballard et al., 2022; Barth et al., 2016; Coenjaerts et al., 2023; De Filippi et al., 2021; Dubol et al., 2021; Fitzgerald et al., 2020; Greenwell et al., 2023; Grotzinger et al., 2024; Hidalgo-Lopez et al., 2021, 2023; Liparoti et al., 2021; Martínez-García et al., 2021; Mueller et al., 2021; Pritschet et al., 2020; Taylor et al., 2020; Zsido et al., 2023), and contribute to the advance knowledge of differential health outcomes for women and people who menstruate.

## METHODS

**Figure 1.**
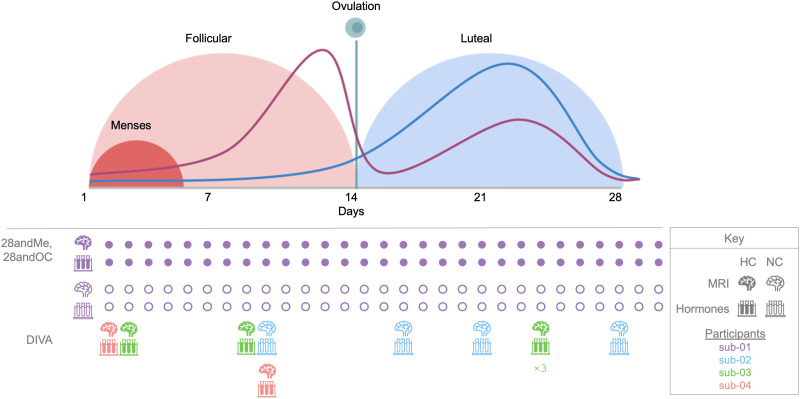
Schematic showing the timing of endocrine and neuroimaging data collection per dataset. The graph (top) is a schematic illustration (i.e., not derived from data presented here) of the phases of the ovarian cycle and standard hormone concentrations throughout. The purple line represents estrogen levels, while the blue line represents progesterone levels (not to scale between hormones). This schematic is based on a 28-day menstrual cycle, which is the average length but not representative of all women. The follicular phase length varies more between individuals, while the luteal phase length is generally more consistent. The timing of data collection for each dataset is presented below. Brain icons indicate days on which MRI data were acquired; test tube icons indicate biospecimen collection days, both of which are color-coded by participant. Filled icons indicate data collected from a participant using HCs, unfilled icons indicate data collected from an NC participant. After the first data points from 28andMe and 28andOC, subsequent data collection days are abbreviated with filled and unfilled circles, respectively, to more simply indicate data collection scheme. While data were collected over the course of a single menstrual cycle for 28andMe and 28andOC, data for DIVA were collected on a weekly resolution and aimed to capture menstrual hormone dynamics over several successive cycles. Cycle day for DIVA participants is based on the self-reported start date of their most recent menses. For sub-03, the resulting data indicate multiple measurements on cycle day 24 (“×3” beneath icons), likely due to a reporting error. Thus, cycle day/phase is not used in any of the following analyses.

Here, we leverage three complementary datasets to assess how hormones and behavior are associated with brain network variability: 28andMe, 28andOC, and DIVA. The sampling scheme for the datasets is shown in Figure 1, with respect to the menstrual cycle, along with a schematic representation of estradiol and progesterone fluctuations across menstrual cycle phases.

### Participants and Study Design

#### 28andMe, 28andOC.

Data were collected from one premenopausal, female participant (monikered “sub-01”; aged 23 years, right-handed) with no history of neurological or psychiatric diagnoses, a history of regular menstrual cycles, and no HC use in the prior year. She completed daily hormone, behavioral, and MRI assessments for 30 days (28andMe) and then again for 30 days 1 year later (aged 24 years), while using HCs (0.02-mg ethinyl-estradiol, 0.1-mg levonorgestrel; Aubra, Afaxys Pharmaceuticals) started 10 months prior (28andOC). Written, informed consent was obtained from this participant before data collection began, in accordance with the University of California, Santa Barbara Human Subjects Committee.

#### DIVA.

Data were collected from three premenopausal, female participants (monikered “sub-02,” “sub-03,” and “sub-04”; age range = 26–31 years). At the time of data collection, two participants were using HCs for at least 6 months and reported no endocrine conditions (sub-04: 0.035-mg ethinyl-estradiol, 0.025-mg norgestimate, Feymor, Amneal Pharmaceuticals; sub-03: 0.02-mg ethinyl-estradiol, 1-mg norethindrone acetate, Blisovi Fe) and the third (sub-02) was freely cycling, with a history of regular menstrual cycles, and had not used HCs in the prior year. Participants completed behavioral assessments and collected saliva samples twice a week, 3–4 days apart, and completed MRI scanning sessions once a week (on a behavioral + hormone collection day). Written, informed consent was obtained from each participant before data collection began, in accordance with Florida International University’s Institutional Review Board approval.

### Endocrine Data

#### 28andMe, 28andOC.

Endocrine measures include serum assessments of gonadotropins (luteinizing hormone and follicle stimulating hormone) and sex steroid hormones 17*β-*estradiol and progesterone concentrations ([E_2_] and [P_4_], respectively). Blood samples (10-ml) were collected daily at or within 30 min of 10:00 a.m. local time. After clotting at room temperature for 45 min and centrifugation at 2,000 g for 10 min, samples were then aliquoted into three 1-ml sterile cryovials and stored at −20 °C until assayed. Liquid chromatography-mass spectrometry was performed at the Brigham and Women’s Hospital Research Assay Core to determine concentrations of serum *β-*estradiol and progesterone.

#### DIVA.

Endocrine measures include salivary [E_2_] and [P_4_] concentrations. Saliva samples were collected via passive drool into 2-ml sterile cryovials shortly after waking twice a week (3–4 days apart) and stored at −20 °C until shipping. Samples were shipped following completion of the study to Salimetrics’ SalivaLab (Carlsbad, CA). To assess [E_2_], samples were assayed using the Salimetrics Salivary Estradiol Assay Kit (Cat. No. 1-3702) with an assay range from 1 to 32 pg/ml, a lower sensitivity limit of 0.1 pg/ml, and a 7.45% interassay coefficient of variation. To assess [P_4_], samples were assayed using the Salimetrics Salivary Progesterone Assay Kit (Cat. No. 1-1502) with an assay range from 10 to 2430 pg/ml, a lower sensitivity limit (5 pg/ml), and a 7.55% interassay coefficient of variation. Samples were assayed in duplicate without modifications to the manufacturers’ protocols. All samples had detectable levels of estradiol, but two samples from sub-03 had no detectable progesterone. The following analyses only use hormone estimates from the same day as MRI scans, to align with 28andMe/28andOC design.

### Neuroimaging Data

#### 28andMe, 28andOC.

MRI data were collected with a 3-Tesla Siemens Prisma scanner equipped with a 64-channel phased-array head coil at the University of California, Santa Barbara. Data include structural T1-weighted images and a 10-min resting-state scan, during which the participant was instructed to keep her eyes open. Structural T1-weighted images were acquired using a T1-weighted MP-RAGE sequence, with TR = 2,500 ms, TE = 2.31 ms, TI = 934 ms, and 0.8-mm slice thickness. Resting-state functional images were collected using a multiband BOLD EPI sequence that acquired 72 oblique slices with TR = 720 ms, TE = 37 ms, a multiband factor of 8, interleaved acquisition, and a 52° flip angle.

#### DIVA.

MRI data were collected with a 3-Tesla Siemens Prisma scanner with a 32-channel head/neck coil at Florida International University’s Center for Imaging Science (Miami, FL, USA). Data used here, collected as part of a larger imaging battery, include structural T1-weighted images and two 5-min resting-state scans, during which participants were instructed to remain still and keep their eyes on a fixation cross presented on a screen outside the bore. Structural T1-weighted images were acquired using a 3D T1w inversion prepared radio frequency-spoiled gradient echo scan, the same sequence used by the Adolescent Brain Cognitive Development^SM^ Study (ABCD Study^®^) (Casey et al., 2018), with anterior-to-posterior phase encoding direction, TR = 2,500 ms, TE = 2.88 ms, TI = 1,070 ms, and 1-mm^3^ isotropic voxels. Resting-state fMRI data were collected using a multiband, multi-echo (MBME) BOLD EPI sequence, from the distribution of multiband accelerated EPI sequences (Moeller et al., 2010) developed by the Center for Magnetic Resonance Research at the University of Minnesota. The MBME gradient echo-EPI sequence acquired 48 slices with 2.4-mm^3^ isotropic voxels at a 30° transverse-to-coronal orientation with anterior-to-posterior phase encoding direction at each of four echoes (TE1 = 11.80 ms, TE2 = 28.04 ms, TE3 = 44.28 ms, TE4 = 60.52 ms) with TR = 1,500 ms, a multiband acceleration factor of 3, interleaved acquisition, in-plane GRAPPA acceleration, and a 77° flip angle.

### MRI Data Processing

Results included in this manuscript are derived from preprocessing performed using fMRIPrep 20.2.1 (Esteban, Blair, et al., 2018; Esteban, Markiewicz, et al., 2018; RRID:SCR_016216), which is based on Nipype 1.5.1 (Gorgolewski et al., 2011, 2018; RRID:SCR_002502). For more detail, see the Supporting Information Methods.

#### Anatomical image preprocessing.

For each participant, T1-weighted images were corrected for intensity nonuniformity using advanced normalization tools, then skull-stripped using OASIS30ANTs as a target template, and segmented into cerebrospinal fluid (CSF), white matter (WM), and GM using FSL’s fast. Nonlinear registration was then performed to align participants’ T1w images with ICBM 152 Nonlinear Asymmetrical template Version 2009c (MNI152NLin2009cAsym).

#### Functional image processing

##### *Single-echo image processing: 28andMe, 28andOC*.

For each BOLD fMRI run, fieldmaps were estimated from EPI reference images with opposite phase encoding directions and then used to create a corrected reference image for coregistration with the participant’s T1w image (6 degrees of freedom, using boundary-based registration). FSL’s motion correction via FMRIB's Linear Image Registration Tool was used to estimate head motion parameters, and slice-timing correction was performed using AFNI’s 3dTshift. Then, bias field correction and motion correction were applied in one step to resample each volume into the participant’s native space. These preprocessed data were then resampled into the MNI152NLin2009cAsym space, and confounding time series (i.e., of head motion, CSF, WM, GM, and their temporal derivatives) were extracted. Data were then high-pass filtered, and principal components of the CSF and WM voxels were extracted (i.e., per aCompCor). Finally, a motion outlier time series was created, flagging any volume with framewise displacement (FD) greater than 0.5 mm.

##### *Multi-echo image processing: DIVA*.

For each BOLD fMRI run, fieldmaps were estimated from EPI reference images with opposite phase encoding directions and then used to create a corrected reference image for coregistration with the participant’s T1w image (6 degrees of freedom, using boundary-based registration). FSL’s MCFLIRT was used to estimate head motion parameters, and slice-timing correction was performed using AFNI’s 3dTshift. Then, bias field correction and motion correction were applied in one step to resample each volume into the participant’s native space. From these data, a quantitative T2* image was estimated via a nonlinear fit to a monoexponential signal decay model using the maximal number of echoes with reliable signal for each voxel. This T2* image was then used to optimally combine the echoes at each TR to create the preprocessed BOLD data. These preprocessed data were then resampled into the MNI152NLin2009cAsym space, and confounding time series (i.e., of head motion, CSF, WM, and their temporal derivatives) were extracted. Finally, a motion outlier time series was created, considering any volume with FD greater than 0.5 mm.

#### Functional connectivity estimation.

Following fMRI preprocessing, the Individual Differences in Connectivity (IDConn, v. 0.3) pipeline was used to estimate connectivity per participant per session (Bottenhorn & Salo, 2024). First, Nilearn was used to mask preprocessed fMRI data with a 268-region atlas generated from via spectral clustering voxelwise resting-state functional connectivity data to define homogeneous, spatially constrained clusters (i.e., regions), covering the cortex, subcortex, and cerebellum (Craddock et al., 2012). After regressing out six motion parameters, CSF and WM signals, and their temporal derivatives, the average residual BOLD signal was extracted per region and standardized. BOLD signals were then correlated, region-wise, to assemble an adjacency matrix per participant per session. Thus, these adjacency matrices represent unthresholded functional connectome estimates as correlation-weighted connections (i.e., edges) between brain regions (i.e., nodes), pairwise, per time point per participant in each dataset.

Participants’ head motion was well within the acceptable range for resting-state fMRI data (i.e., mean FD < 0.2 mm; Supporting Information Table S1), indicating that no participant’s imaging data were sufficiently corrupted by motion. While head motion did differ between the two datasets, it was unrelated to functional connectivity, HC use, [E_2_], or [P_4_] (Supporting Information Table S2).

### Inferential Statistics

#### Missing data across variables.

First, we assessed missingness per variable by calculating (a) the proportion of missing values per participant per variable and (b) point-biserial correlation coefficients between missingness on each variable and the values of each other variable to determine whether variables were missing at random or not.

Missing data were < 10% per participant per variable, and there was no evidence to suggest missingness on any one variable was conditioned on values of other variables, providing no sufficient evidence that missingness is not completely at random.

#### Analysis 1: Connectome similarity.

The degree of functional connectome similarity (i.e., across the whole brain) due to each (a) group (i.e., shared across participants), (b) dataset, (c) HC use status (i.e., HC using or NC), and (d) individual was quantified by computing a product-moment correlation coefficient for each pair of connectomes (i.e., the upper triangle of the adjacency matrix). Then, the Fisher transformation was used to convert these correlation coefficients to *z* scores. Connectome similarity was calculated as the average of Fisher-transformed product-moment correlations (a) between different individuals and sessions (i.e., *group*-level similarity), (b) between individuals in the same *dataset*, (c) between individuals with the same *HC use* status, and (d) between all sessions from each *individual*, respectively. The connectome similarity values were then compared between each of the subdivisions using two-sample *t* tests, evaluated at a significance threshold of *α* < 0.01.

#### Analysis 2: Hormone-related connectomics.

**Figure 2.**
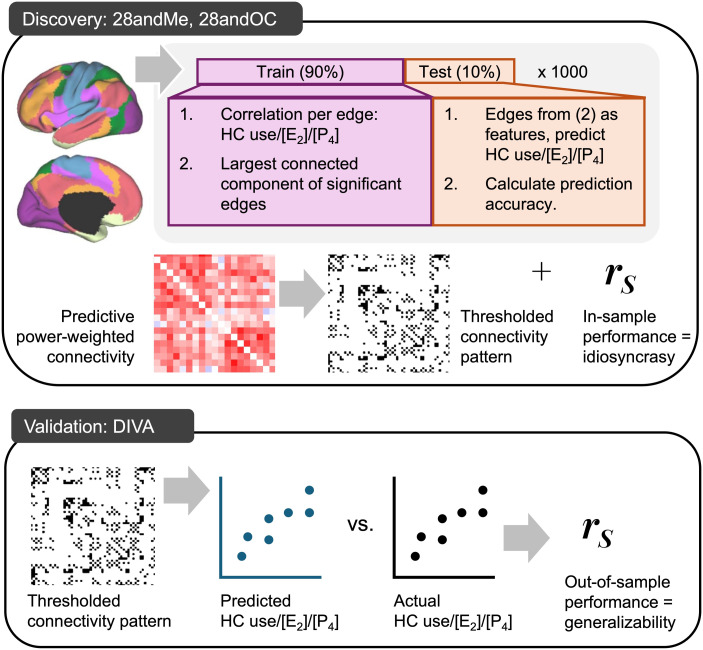
Illustration of the multidataset predictive connectomics approach used here to identify contraceptive- and hormone-related connectomics. First, data from 28andMe + 28andOC (one participant, 60 sessions) was used to identify connectivity related to HC use, [E2], and [P4], in turn (top: Discovery). In a 10-fold cross-validation framework, 90% of the data (i.e., 54 sessions), each edge (i.e., connection) from the 268 × 268 connectivity matrices was correlated with the outcome variable (i.e., HC use, [E2], or [P4]), and then, for the weak type-II error rate control, the edges comprising the largest connected component of significant correlations were used as features in a kernel ridge regression (KRR) to predict values of the outcome variable in the held-out 10% of the data (i.e., six sessions). This process was repeated 1,000 times and yielded a connectivity matrix of outcome-related edges, weighted by the predictive performance of models in which they were included, in addition to estimates of in-sample performance, a proxy for idiosyncrasy. This weighted matrix was thresholded to identify the edges/connections to be used as features in the next analyses. Second, data from DIVA (three participants, 10 total sessions) were used to validate the identified patterns of contraceptive- and hormone-related connectivity. Edges from the thresholded connectivity pattern were used as KRR features to predict values of the outcome variables (i.e., HC use, [E2], or [P4]), based on connectivity values from DIVA data. Comparing these predicted values with actual values from DIVA (i.e., hormone levels from saliva samples collected on the same days as MRI scans) yielded a measure of out-of-sample performance, which is a proxy for the generalizability of the identified hormone- and contraceptive-related connectivity patterns.

Finally, we used a predictive modeling approach to identify intrinsic functional connectivity related to HC use and within-individual differences in hormone levels across the menstrual and HC pill cycles and then applied transfer learning to test their generalizability out-of-sample (i.e., between individuals; Figure 2).

First, a Python adaptation of NBS-Predict (Serin et al., 2021), included in IDConn (Supporting Information Figure S1), was used to identify functional connectivity related to HC use in a fivefold cross-validation framework with hyperparameter tuning to determine the optimal *L*_2_ coefficient (i.e., *α*). To do so, data from 80% of the time points (i.e., 48 sessions) in 28andMe and 28andOC were used to train a model, following within-fold regression of average head motion from each edge, by (a) performing connection-wise *F* tests for connectivity differences between NC- and HC-using timepoints and (b) selecting functional connections that were significantly associated with the HC use above a given *p* threshold (i.e., *p*(*F*) < 0.05). From these suprathreshold connections, (c) NBS-Predict determined the HC-associated connectivity, ***N***_***HC***_, as the largest connected component of suprathreshold connections in which each brain region is connected to every other brain region either directly or indirectly, removing the connections that were not part of that component. The strengths of these connections were, then, used as features in a logistic regression model of the form in Equation 2, regressing HC use onto connectivity in the remaining 20% of time points (i.e., 12 sessions), to estimate how related the connectivity pattern as a whole is to HC use.

$$HC\;use=\beta {FC}_{HC}+FD+\epsilon$$ (1)

This process was repeated 1,000 times, randomly sampling the training dataset (28andMe + 28andOC) to yield a distribution of HC-related connectivity and its accuracy in classifying HC use. Then, an average of these connections, weighted by the classification accuracy of models in which they are included, was calculated and thresholded to determine the functional connectivity pattern most associated with HC use. Finally, this connectivity was used to train a logistic regression model on the full training dataset (i.e., all 60 sessions from 28andMe + 28andOC) and the resulting model parameters (i.e., *α, β*) were used to predict HC use from connectivity in DIVA data and estimate generalizability from out-of-sample predictive accuracy.

Second, NBS-Predict was similarly used to separately predict [E_2_] and [P_4_] from functional connectivity in a fivefold cross-validation framework, by (0) regressing HC use and FD from [E_2_], [P_4_], and each edge, (1) calculating connection-wise *F* tests between the residualized connectivity and [E_2_] and [P_4_] in 80% of 28andMe and 28andOC time points (i.e., hormone-related connectivity accounting for HC use) to (2) select suprathreshold connections (i.e., *p*(*F*) < 0.05) and (3) identify separate estradiol- and progesterone-associated connectivity, *N*_*E2*_ and *N*_*P4*_, from the largest connected components. Then, linear regression with *L*_2_ regularization (i.e., ridge regression) was used to predict the hormone levels, per Equation 3, in the remaining 20% of 28andMe and 28andOC data. Model performance was assessed via correlations between actual and predicted hormone concentrations, per NBS-Predict methodology (Serin et al., 2021), but using Spearman’s correlation instead of product–moment correlations to accommodate unmet distributional assumptions. Each model was also run without regressing out HC use (see parentheticals in Equations 2–3), to examine the role of exogenous hormones on connectivity related to endogenous hormones, in this context.

$$\left[E_{2}\right]=\beta {FC}_{E2}\left(+HC\;use\right)+FD+\epsilon$$ (2)

$$\left[P_{4}\right]=\beta {FC}_{P4}\left(+HC\;use\right)+FD+\epsilon$$ (3)

As above, this process was repeated 1,000 times and the resulting connections and Spearman correlations were used to assemble weighted average estradiol- and progesterone-associated connectivity patterns, weighted by model performance. These connectivity patterns were then thresholded, retaining 50% of the most positively outcome-related edges, and applied to the full training dataset to train linear regression models for each [E_2_] and [P_4_]. The resulting model parameters (i.e., *α, β*) were then used to predict [E_2_] and [P_4_] from DIVA connectivity data, to determine their out-of-sample generalizability.

Finally, we ran post hoc sensitivity analyses, dropping the high [E_2_] days (i.e., ovulatory peaks, menstrual cycle days 12–16) from the data used to identify HC- and hormone-related connectivity patterns (i.e., training dataset: 28andMe, 28andOC, as those days were not sampled in the DIVA dataset and brain–hormone associations vary across menstrual cycle phases; Casto et al., 2022).

## RESULTS

**Figure 3.**
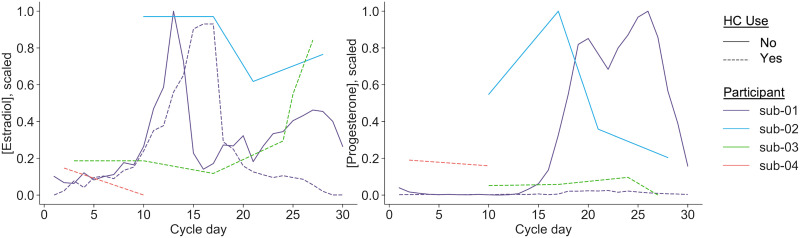
Dataset-scaled estradiol and progesterone concentrations across the sample. Estradiol (left) and progesterone (right) were assessed via assay of either saliva or blood samples, as the collection method differed between datasets. Shown here are estradiol and progesterone concentrations per participant across the menstrual cycle, scaled between 0 and 1 within each dataset (i.e., 28andMe/28andOC; DIVA). Solid lines indicate freely cycling participants (i.e., sub-01, sub-03), while dashed lines indicate participants using HCs (i.e., sub-01, sub-02, sub-04). Variations in length and reporting of menstrual cycle cause lags between estradiol and progesterone increases seen in menstruating women. Cycle day for DIVA participants is based on the self-reported start date of their most recent menses. For sub-03, the resulting data indicate multiple measurements on cycle day 24 (see Figure 1), likely due to a reporting error. Thus, cycle day for sub-03 is extrapolated in this plot and cycle day, in general, is not used in any of the following analyses.

Hormone concentrations across the measurement periods (scaled between 0 and 1, within each dataset, for comparison across measurement techniques) are displayed in Figure 3, showing differences in cycle timing and measurement period across subjects. Here, “cycle day” refers to the number of days since the start of the participant’s most recent menses.

### Analysis 1: Connectome Similarity

**Figure 4.**
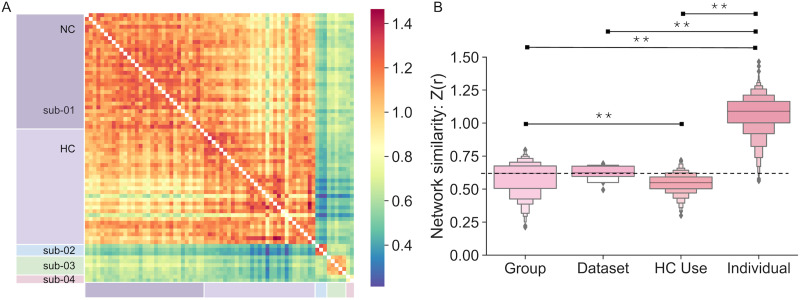
Network similarity across participants and sessions. (A) Network similarity is shown as correlation coefficients Fisher-transformed for normality (*Z*(*r*)). Rows/columns are sorted by participant and session. First, sub-01 sessions while NC (in 28andMe) and while using HCs (in 28andOC) are delineated, then sub-20, sub-03, and sub-04 from DIVA are shown. (B) Network similarity is calculated across the group (from different individuals, datasets, HC use strategies), between datasets (from different individuals and HC use strategies), across HC use strategies (from different individuals and datasets), and within individuals (from different sessions). Asterisks indicate significant differences in network similarity, *p* < 0.01.

Overall, networks were more similar within individuals than between individuals (Figure 4A). In line with results from Gratton et al. (2018), we found that network similarity (Figure 4B) within each individual was more than twice the magnitude of similarity between individuals (i.e., across the group). Additionally, we found that network similarity was of similar magnitude between dataset and HC use status, although group-level similarity was significantly greater than similarity based on HC use status (*p* < 0.01). However, as one dataset (i.e., 28andMe, 28andOC) was limited to one individual, these results should be interpreted with caution and warrant further investigation in a larger multisite dataset.

### Analysis 2: Contraceptive- and Hormone-Related Connectomics

NBS-Predict revealed a diffuse, brain-wide connectivity pattern associated with HC use, as well as more limited [E_2_]- and [P_4_]-related connectivity patterns, with varying generalizability to independent data (Table 1).

**Table 1.** Performance and generalizability of contraceptive- and hormone-related connectivity patterns

	HC use	Estradiol		Progesterone	
		Base	HC use	Base	HC use
CV mean ± *SD*	79.8% ± 11.4%	0.130 ± 0.277	0.025 ± 0.304	0.357 ± 0.252	0.189 ± 0.296
Alpha	5.99 × 10^−3^	7.74 × 10^−4^	5.99 × 10^−3^	1.00 × 10^−4^	5.99 × 10^−3^
In-sample (28andMe/28andOC)					
Model performance	74%	0.937	0.814	0.804	0.950
MSE	0.0833	0.174	0.368	0.0861	0.0172
Out-of-sample (DIVA)					
Model performance	50%	0.182	0.648	0.429	−0.357
MSE	0.5	0.619	0.0810	0.0719	0.118

*Note*. HC use models are logistic regression with *L*_2_ penalties, and their performance is percent accuracy of HC use predicted by functional connectivity. Estradiol and progesterone models are linear regression with *L*_2_ penalties (i.e., ridge regression) and their performance is provided by the Spearman correlation between true hormone concentrations and hormone concentrations predicted from functional connectivity. Results from sensitivity analyses are shown in the Supporting Information Table S3.

#### Contraceptive-related functional connectivity changes.

**Figure 5.**
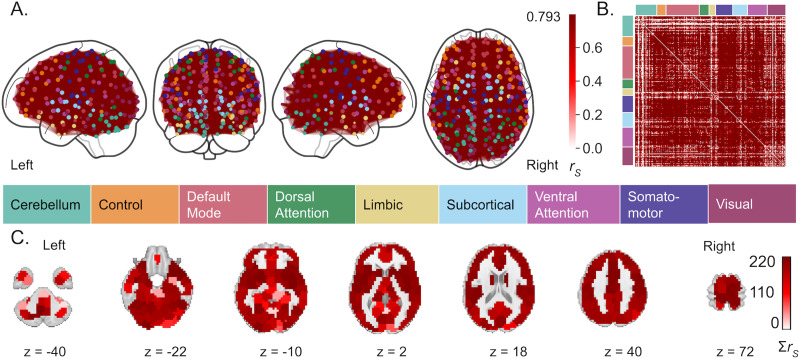
Functional connectivity predictive of HC use. (A) The top 0.001% of functional connections most predictive of HC use across training folds. Node color is determined by the functional network, as defined by Yeo et al. (2011) with which each region demonstrates the greatest overlap. Edge color is determined by the average accuracy of the identified connectivity pattern in predicting HC use in the held-out sample, across training folds, reflecting the relative importance of each connection to the overall pattern but not an effect size. Average accuracy was consistently high, that most edges shared the same, maximum value (i.e., 0.799). (B) Average accuracy of all functional connections in predicting HC use across model training. (C) HC use-associated node strength, or the sum of each brain region’s accuracy-weighted functional connections, with *z*-coordinates of each slice given in Montreal Neurological Institute space.

Within-individual contraceptive-related functional connectivity was robust and included nearly every region of the brain (Figure 5). Average model accuracy across model-building iterations (i.e., assessed on an unseen subset of 28andMe, 28andOC data) was 79.8%, and overall in-sample accuracy was 74%. When generalizability of the contraceptive-related connectivity pattern was assessed on an independent sample (i.e., DIVA), accuracy was 50%. There was little variation, spatially or between model-building iterations, in the predictive power of functional connections across the brain (Figure 5A–B). Regionally, the insula, caudate, midcingulate cortex, superior postcentral gyri, and orbitofrontal cortex displayed the greatest contraceptive-related connectivity, while the cerebellum and medial temporal lobe displayed the least. Across large-scale brain networks, somatomotor and ventral attention networks (VAN) displayed the greatest contraceptive-related connectivity, while cerebellar, limbic, and visual networks displayed the least (Supporting Information Figure S2, top row).

#### Hormone-related functional connectivity.

**Figure 6.**
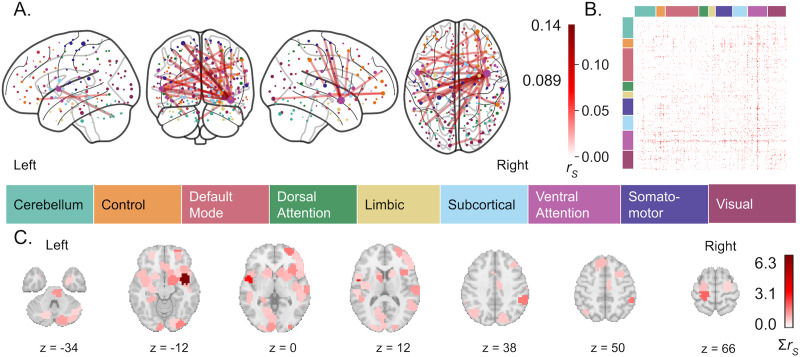
Functional connectivity predictive of estradiol levels. (A) The top 0.15% of functional connections most predictive of [E_2_] across training folds. Node color is determined by the functional network, as defined by Yeo et al. (2011) with which each region demonstrates the greatest overlap. Edge color is determined by the average Spearman correlation (i.e., *r*) of predicted [E_2_] versus actual [E_2_] across training folds, reflecting the relative importance of each connection to the overall pattern but not an effect size. (B) Weighted average connectivity, using Spearman correlation (i.e., *r*) of model’s predicted [E_2_] versus actual [E_2_] across training folds. (C) [E_2_]-associated node strength, or the sum of each brain region’s *r*-weighted functional connections, with *z*-coordinates of each slice given in MNI space.

Estradiol-related functional connectivity was sparser (Figure 6; Supporting Information Figure S1, middle row). Average Spearman correlation between actual and predicted estradiol concentrations, within 28andMe, 28andOC data, was 0.130 (±0.277) without HC use as a covariate and 0.025 (±0.304) with HC use as a covariate. Thus, accounting for HC use caused the identified connectivity pattern to less accurately describe [E_2_] in the held-out sessions, but with a wider spread, potentially indicating more variability in the edges identified as related to [E_2_]. Once the performance-weighted connectivity was thresholded to only include the top 50% of [E_2_]-related connections (i.e., with average *r* > 0 across training folds), overall in-sample correlation was 0.168 without HC use as a covariate and 0.0392 with HC use as a covariate. When the [E_2_]-relatedness of the same connectivity was assessed in an independent dataset (i.e., DIVA), out-of-sample correlation was −0.491 without HC use as a covariate and 0.648 with HC use as a covariate. Thus, the identified functional connectivity pattern demonstrated moderate performance in estimating [E_2_] within an individual; its generalizability to other individuals depended on HC use. When accounting for HC use, the identified connectivity pattern performed worse within an individual, but better in independent data. Neuroanatomically, the right anterior insula demonstrated the highest node strength in the [E_2_]-related connectivity pattern (Figures 6A and 6C). Accounting for HC use, estradiol-related connectivity was greatest in VAN regions, most notably in the anterior insula and between VAN and somatomotor regions. In contrast, connectivity of virtually no dorsal attention, limbic, or subcortical regions were related to changing [E_2_] (Supporting Information Figure S2, middle row).

**Figure 7.**
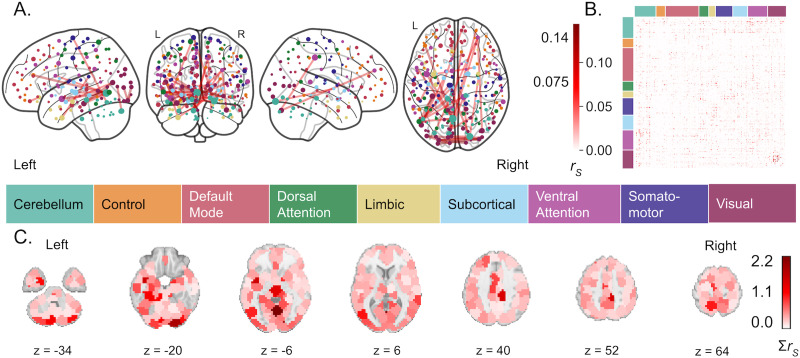
Functional connectivity predictive of progesterone levels. (A) The top 0.15% of functional connections most predictive of [P_4_], across training folds, accounting for HC use. Node color is determined by the functional network, as defined by Yeo et al. (Yeo et al., 2011) with which each region demonstrates the greatest overlap. Edge color is determined by the average Spearman correlation (i.e., *r*) of predicted [P_4_] versus actual [P_4_] across training folds, reflecting the relative importance of each connection to the overall pattern but not an effect size. (B) Weighted average connectivity, using Spearman correlation (i.e., *r*) of model’s predicted [P_4_] versus actual [P_4_] across training folds. (C) [P_4_]-associated node strength, or the sum of each brain region’s *r*-weighted functional connections, with *z*-coordinates of each slice given in MNI space.

Compared with estradiol, progesterone-related functional connectivity was considerably denser and more widespread (Figure 7). The final model’s Spearman correlation between actual and predicted serum progesterone concentrations (i.e., tested on an unseen subset of 28andMe, 28andOC data) was 0.80 (*p* < 0.01; mean squared error: 0.086) without HC use as a covariate and 0.95 (*p* < 0.01; MSE: 0.0172) with HC use as a covariate. Thus, accounting for HC use caused the identified connectivity pattern to more accurately reflect changing [P_4_], potentially due to a confounding blunting of progesterone levels by HC use. Throughout the network-identification process, adding HC use yielded poorer performance and a greater range of model fit, indicating greater variability in the identified networks. The final connectivity pattern, however, was a more accurate reflection of progesterone-related functional connectivity in that individual when accounting for HC use, but showed less generalizability to other individuals, as shown by greater error in [P_4_] estimated from others’ functional connectivity (without HC: *r* = 0.49, MSE: 0.0719; with HC: *r* = −0.357, MSE = 0.118). Unlike with [E_2_]-related functional connectivity, adjusting for HC use caused the identified [P4]-related functional connectivity pattern’s estimation of hormone levels in novel data to perform worse than did the connectivity pattern identified when not adjusting for HC use. Accounting for HC use, the progesterone-related connectivity pattern demonstrated the most connectivity between cerebellar and ventral attention regions, within the visual network, and between limbic regions and each subcortical, dorsal attention, and visual regions. In contrast, other visual connectivity, frontoparietal control network connectivity, and default mode network connectivity were less related to changing [P_4_] over the menstrual cycle (Supporting Information Figure S2, bottom row).

#### Sensitivity analyses: Removing ovulatory peaks from training dataset.

We repeated all predictive connectomics analyses after excluding time points from the training dataset that fell within the ovulatory window (Supporting Information Table S3). Without the high-estradiol days within the ovulatory window, HC-related connectivity exhibited poorer in-sample model fit and worse generalizability to DIVA Study data (i.e., 50% accuracy). Compared with the models presented above, the [E_2_]-related connectivity pattern identified without ovulatory window data exhibited better in-sample model fit when accounting for HC use, worse in-sample model fit when not accounting for HC use, and poorer generalizability in both cases. Compared with the models presented above, the [P_4_]-related connectivity pattern identified without ovulatory window data, on the other hand, demonstrated worse in-sample performance when accounting for HC use, but better in-sample model fit when not accounting for HC use, and better generalizability to DIVA data regardless of accounting for HC use. The topography of HC-related connectivity identified without ovulatory window data was virtually identical to the connectivity pattern presented above: widespread, highly related connectivity. The topography of the [E_2_]- and [P_4_]-related connectivity (i.e., with and without accounting for HC use) were largely unchanged following the removal of ovulatory window data (Supporting Information Figure S3).

## DISCUSSION

Steroid hormones are critical neuromodulators. As human neuroimaging strives toward population neuroscience and precision medicine, understanding hormones’ roles in brain function, as well as idiosyncrasies and commonalities therein, becomes paramount. First, these results replicate prior findings from Gratton and colleagues (2018): Stable features common across the group and specific to individuals contribute almost equally to functional connectome similarity. Furthermore, we extend this work to show that individual-specific features continue to contribute significantly to connectome similarity, beyond differences in datasets and participant HC use. Second, predictive modeling identified widespread functional connectivity changes associated with HC use, and sparser changes related to estradiol and progesterone fluctuations throughout the menstrual cycle. The identified HC- and hormone-related connectivity exhibited mixed generalizability to other individuals, potentially reflecting differential brain–hormone associations depending on HC progestin formulations. Together, this work marks an important step forward in understanding neuroendocrine dynamics inherent to the adult brain in people who menstruate and begs further study of contraceptive-related differences and functional connectomics across the menstrual cycle.

### Individual-Specific Functional Connectivity

The brain’s functional network organization is relatively idiosyncratic from birth (Wang et al., 2021) into adulthood (Gratton et al., 2018) in both its neuroanatomy (Seitzman et al., 2019) and connectomics (Finn et al., 2015). Here, we replicate prior findings regarding approximately equal influences of group commonalities and individual-specific factors on functional connectomics (Gratton et al., 2018). Using data from two independent datasets with adult female participants of similar age (i.e., 23–31 years), we found that the magnitude of contributions of dataset differences and imaging sequence (inextricable in these data) to network similarity are not significantly different than that of group commonalities, although HC use contributes slightly, but significantly less, than do group commonalities. Even in the face of a common exogenous hormone manipulation from HC use, individual traits dominate functional connectivity. The relative contributions of HC use and individual factors to functional connectivity are particularly interesting on two fronts. First, there is evidence that the menstrual cycle varies as much within individuals as between, which neatly aligns with our findings. Following the approach described by Gratton et al., the “normalized effect magnitude” can be used to compare relative contributions of each variable by subtracting connectome similarity *z* scores. Thus, after accounting for similarity due to shared group factors (*z* = 0.59), the normalized effect magnitude of individual-specific factors indicates a similar relative contribution of group and individual factors (i.e., 1.07 − 0.59 = **0.48**). Second, the observed ubiquity of within-individual HC-related functional connectivity changes, in contrast with near-chance accuracy in predicting HC use in other individuals, aligns with the relative effect magnitudes of group-shared versus individual-specific factors and HC use versus other individual-specific factors (i.e., 1.07 − 0.54 = **0.53**). That is, although we found that exogenous hormone manipulation via HC use is robustly related to brain-wide changes in connectivity within an individual, HC use may be no more distinctive than other individual-specific factors.

### Contraceptive-Related Changes in Functional Connectivity Are Ubiquitous

HC use impacted functional connectivity of every large-scale functional brain network, with the smallest impacts in cerebellar, visual, and limbic regions and the largest impacts in dorsal and ventral attention networks as well as frontoparietal control, somatomotor, and default mode networks. As these connectivity patterns were identified using data from only one individual, their generalizability to other individuals provides additional information about the extent to which individual-specific traits inform neuroendocrine fluctuations during the adult menstrual cycle and the extent to which HCs affect these fluctuations. Two notable findings arose from assessing generalizability of the identified endocrine-related connectivity. First, accounting for HC use in models identifying hormone-related connectivity improved our ability to identify progesterone-related connectivity but worsened our ability to identify estradiol-related connectivity. Second, and conversely, accounting for HC use in uncovering hormone-related connectivity improved the generalizability of estradiol-related connectivity but substantially worsened the generalizability of progesterone-related connectivity. Although these results represent many observations from only a few individuals, they recapitulate prior findings regarding the extent to which HC use alters functional connectivity, including diffuse, altered connectivity between several large-scale brain networks (Casto et al., 2022; Petersen et al., 2014).

Differences in model fit due to accounting for HC use indicate that estradiol- and progesterone-related connectivity is differentially confounded by the exogenous hormones in oral contraceptives. However, accounting for HC use yielded an estradiol-related connectivity pattern *more* robust to individual differences but rendered the progesterone-related connectivity pattern *less* robust. The participants in these studies all used HCs with different progestin formulations and different amounts of ethinyl estradiol, which makes it difficult to disentangle individual-specific and formulation-related differences. The diminished generalizability of progesterone-related functional connectivity may reflect differential effects on functional connectivity between progestins. This possibility is bolstered by the fact that the progestins in each HC formulation used by participants in this study have different pharmacological and neurobiological effects (Bullock & Bardin, 1977; Casto et al., 2022; Griksiene et al., 2022). In addition to their progestational effects, norethindrone acetate (used by sub-03 in DIVA) has strong androgenic but weak estrogenic effects, norgestimate (used by sub-04 in DIVA) has androgenic effects and antiestrogenic effects, and levonorgestrel (i.e., used by sub-01 in 28andOCs) has high androgenic effects, but weak estrogenic effects (Griksiene et al., 2022). Further research including a larger sample size with measurements of synthetic hormones (e.g., ethinyl estradiol, levonorgestrel) in multiple, independent datasets would be required to support this theory and clarify the interactions between exogenous, synthetic hormones from HCs and endogenous hormones.

### Hormone-Related Functional Connectivity Changes Across the Menstrual Cycle

We identified estradiol- and progesterone-related connectivity that covary with hormone levels across the menstrual cycle, using 2 months of daily biospecimen and functional neuroimaging data. Estradiol-sensitive connectivity was dominated by ventral attention, visual, and cerebellar network regions with core connections of bilateral anterior insula, occipital, orbitofrontal, (para)hippocampal, and cerebellar connectivity that persisted when accounting for HC use. Progesterone-sensitive connectivity, however, was more widespread. Accounting for HC use affected core progesterone-related connections, diminishing connectivity of midline frontal and precuneus regions while increasing within-occipital lobe connectivity. Robustly identifying progesterone-related functional connectivity is further complicated by unmeasured levels of progesterone synthesized in the brain that are distinct from circulating progesterone (measured here) and modulated by estradiol (Micevych & Sinchak, 2008).

Much of the extant literature describing brain changes along the menstrual cycle lacks methodological rigor and statistical power (Dubol et al., 2021), making it difficult to reliably contextualize these findings, as does this study’s small sample and demonstrated connectome idiosyncrasy. Some have identified no effects of menstrual cycle fluctuations on resting-state functional connectivity (De Bondt et al., 2015; Hjelmervik et al., 2014), although this may be due to insufficient statistical power with 16–18 NC female participants and only 3 time points each (i.e., once per menstrual cycle phase). However, a few consistent findings align with our results. A recent dynamic causal modeling study scanned 60 NC women three times (i.e., in early follicular, pre-ovulatory and mid-luteal phases) and uncovered estradiol-related insula connectivity with hemispheric differences, as seen here (Hidalgo-Lopez et al., 2021). A recent meta-analysis identified convergent GM volume and task-based activation differences in the insula over the course of the menstrual cycle (Dubol et al., 2021). Prior functional connectivity studies have also identified estradiol-related connectivity of occipital, inferior frontal, and temporal cortex regions (Craig et al., 2008; Engman et al., 2016; Meeker et al., 2020). Interestingly, after accounting for HC use in the current study, estradiol-related functional connectivity involved relatively little limbic and subcortical connectivity, despite their relatively high estrogen receptor expression (i.e., ER*α* and, to a lesser extent, ER*β*; Österlund, Gustafsson, et al., 2000; Österlund, Keller, et al., 2000) and estradiol concentrations (Bixo et al., 1995). This may be due to shared variance in subcortical–limbic connectivity between exogenous and endogenous estradiol levels, but it is unlikely given that subcortical and limbic regions play a relatively small role in the identified HC-related connectivity pattern. Thus, there may be interaction effects between estradiol and HC actions in subcortical and limbic connectivity that warrant future study. Finally, our results did not include the expected estradiol-related PFC connectivity. Instead, we found a minimal role for PFC connectivity in the estradiol-related connectivity pattern, regardless of accounting for HC use. Prior research indicates that PFC connectivity changes between the luteal and follicular phases of the menstrual cycle (Engman et al., 2018; Meeker et al., 2020; Wetherill et al., 2016), which may explain why it was not revealed here, as we did not incorporate menstrual cycle phase in these models.

### Limitations and Future Directions

Incorporating multiple datasets with different sampling rates and data collection methods has both strengths and limitations. Data in 28andMe and 28andOC were collected from a single participant, separated by 1 year with a 10-month HC wash-in period. Thus, in training the models, elapsed time, and HC use cannot be entirely disambiguated. Thus, the observed HC-related functional connectivity may also be related to differences in brain function seen over a year. This effect of time likely contributes to the poor generalizability of the identified HC-related connectivity pattern and is at least partially accounted for in the hormone models that account for HC use. The other consequence of study design is that these data cannot be used to address lagged associations between hormone concentrations and functional connectivity (as previously demonstrated by Pritschet et al., 2020), as DIVA Study participants only have biweekly, salivary hormone estimates and weekly MRI data. Furthermore, these results only represent a small proportion of potential HC-related functional connectivity and HC-related alterations of hormone-related functional connectivity, as only three HC formulations are represented in these data with one formulation per participant. Future research should include and compare oral contraceptive formulations (e.g., androgenic vs. antiandrogenic progestins, progestin-only mini pill), as well as other forms of HCs (e.g., implant, shot, intrauterine devices), while measuring concentrations of both exogenous and endogenous hormones.

In contrast to other multidataset investigations, we did not perform neuroimaging data harmonization between 28andMe, 28andOC, and DIVA Study data. In using independent datasets for model development and validation, we chose not to harmonize the neuroimaging data to avoid data leakage and any resulting bias of model coefficients or performance (Rosenblatt et al., 2024). Thus, our results regarding out-of-sample model performance reflect the generalizability of the identified contraceptive- and hormone-related connectivity patterns *despite* differences in MRI sequences and scanners, as well as differences in hormone estimates (i.e., salivary vs. serum), experimental design, and other confounding factors (e.g., time of day, temperature, air quality, season). While salivary and serum estimates of progesterone are generally highly correlated (Ellison, 1993), associations between salivary and serum estimates of estradiol can vary between individuals, potentially due to differences in salivary gland function (Lu et al., 1999) and procedures for estimating estradiol from saliva (e.g., immunoassays) are less reliable than those used to estimate estradiol from serum samples (e.g., liquid chromatography mass spectrometry; Newman & Handelsman, 2014). Generalizability of estradiol-related connectivity identified here may be influenced by these differences in hormone estimation.

Additionally, these analyses do not address the potential of a changing brain–hormone relationship across the menstrual cycle, as has been identified previously (Syan et al., 2017). The limited data available are insufficient to address different brain–hormone associations between menstrual cycle phases. Interestingly, 28andMe and 28andOC included brain and hormone measures collected during the high-estradiol ovulatory window, but the DIVA Study did not, and when ovulatory window data were removed in sensitivity analyses, both [E_2_]- and [P_4_]-related networks were topographically unchanged but [E_2_]-related connectivity showed poorer generalizability, while [P_4_]-related connectivity showed greater generalizability. Prior work with the 28andMe data showed that high-estradiol days during the ovulatory window were driving their estradiol-related connectivity findings (Pritschet et al., 2020). The marginally worse generalizability of these models in a dataset that did not measure the high-estradiol ovulatory window is curious. Future work with larger samples, and more within-individual data across menstrual cycle phases than is available in the DIVA Study, will help better understand the impact of cycle phase and ovulatory estradiol peaks on hormone-related connectivity.

More broadly, this work contributes to a growing literature on the rapid neuromodulatory potential of sex hormones (Grotzinger et al., 2024; Pritschet et al., 2021; Taylor et al., 2020; Zsido et al., 2023). Thus, we would recommend that more neuroimaging studies prioritize collecting data on participants’ hormone cycles and, where possible, hormone levels. Such data are relevant for studying or accounting for fluctuations across the menstrual cycle and across diurnal/circadian rhythms (Grotzinger et al., 2024). Thus, unmeasured hormone levels likely represent a notable confounding factor or detriment to precision for many research questions. This is especially important for large-scale cohort studies that have sufficiently large samples to address individual differences in neuroendocrine phenomena and sufficiently broad measures to account for neuroendocrine confounds. To account for menstrual cycle differences, researchers should consider collecting, at a minimum, participant-reported menses start dates and, ideally, biospecimens for hormone assessments (Schmalenberger et al., 2021). To account for diurnal or circadian rhythm differences, researchers should consider collecting, at a minimum, participant-reported sleep timing and the time of day that MRI data were acquired, in addition to, ideally, biospecimens for hormone assessments (Van Cauter, 2008). While analyzing biospecimens can become prohibitively expensive, observed hormonal effects in brain structure and function can substantially confound other brain-phenotype associations (e.g., endocrine modulation of hippocampal volume likely confounds learning and memory research).

### Conclusions

Here, we used two densely sampled longitudinal datasets to assess individual-specific features of the functional connectome; to identify contraceptive-, estradiol-, and progesterone-related functional connectivity across the menstrual cycle; and to assess the generalizability of those connectivity patterns. Our findings corroborated prior work regarding the balance of common and individual-specific contributions to functional brain organization, while extending the literature to consider the contribution of HC use. Furthermore, we identified brain-wide changes in functional connectivity associated with HC use, which may be more idiosyncratic than they are generalizable, and smaller hormone-related connectivity patterns. Interestingly, accounting for HC use improved the generalizability, but not accuracy, of estradiol-related connectivity, but had the opposite effect on progesterone-related connectivity. Together, these findings hint at individual and progestin-related differences in functional brain network organization across the menstrual cycle but underscore the need for larger studies of contraceptive-related changes in brain function with sufficient statistical power to compare potential effects of different HC formulations. As the study of human neuroendocrinology advances with dense sampling and precision approaches, it is imperative that women not be left behind.

## Acknowledgments

Research described in this article was supported by NIH U01-DA041156 (A.R.L., K.L.B.), R01-ES032295 (M.M.H., K.L.B.), R01-ES031074 (M.M.H., K.L.B.), and K99-MH135075 (K.L.B.). Additional support was provided by the Office of Research and Economic Development and the Dissertation Year Fellowship Program at Florida International University. Finally, the authors would like to thank the FIU Instructional & Research Computing Center (https://ircc.fiu.edu) for providing the HPC and computing resources that contributed to the research results reported within this paper.

## Supporting Information

Supporting information for this article is available at https://doi.org/10.1162/netn.a.20.

## Author Contributions

Katherine L. Bottenhorn: Conceptualization; Data curation; Formal analysis; Investigation; Methodology; Project administration; Software; Visualization; Writing – original draft; Writing – review & editing. Taylor Salo: Data curation; Formal analysis; Investigation; Methodology; Software; Writing – review & editing. Emily G. Jacobs: Resources; Writing – review & editing. Laura Pritschet: Data curation; Writing – review & editing. Caitlin Taylor: Writing – review & editing. Megan M. Herting: Supervision; Writing – review & editing. Angela R. Laird: Conceptualization; Funding acquisition; Methodology; Project administration; Supervision; Writing – original draft; Writing – review & editing.

## Funding Information

Angela R. Laird, National Institute on Drug Abuse (https://dx.doi.org/10.13039/100000026), Award ID: U01DA041156. Megan M. Herting, National Institute of Environmental Health Sciences (https://dx.doi.org/10.13039/100000066), Award ID: R01ES032295. Megan M. Herting, National Institute of Environmental Health Sciences (https://dx.doi.org/10.13039/100000066), Award ID: R01ES031074. Katherine L. Bottenhorn, National Institute of Mental Health (https://dx.doi.org/10.13039/100000025), Award ID: K99MH135075.

## Supplementary Material



## TECHNICAL TERMS

Progesterone: Sex steroid hormone, the most prevalent progestin in the human body, involved in sexual differentiation and reproduction.
Endogenous: Referring to hormones, produced by the body.
Exogenous: Referring to hormones, produced outside the body and ingested (e.g., hormonal contraceptives).
Generalizability: Applicability of scientific results (here, contraceptive- and hormone-related connectivity patterns) in a broader context or to a broader sample.
Idiosyncrasy: A feature or characteristic (here, functional connectivity pattern) that is specific, or unique, to one individual.
Dense sampling: A longitudinal data collection scheme that involves acquiring data from individuals at a relatively high sampling rate (e.g., daily, weekly).
17*β*-estradiol: Sex steroid hormone, the most prevalent estrogen in the human body.
BOLD fMRI: Blood-oxygen-level-dependent fMRI, uses paramagnetic properties of oxygenated blood to estimate brain activity and functional connectivity.
Hormonal contraceptive formulation: Referring to combined oral contraceptives (i.e., birth control pills), the specific combination and concentrations of synthetic estrogen and progestin.
Progestational: Central nervous system actions of progestins (e.g., progesterone, norgestimate, norethindrone acetate, levonorgestrel).
Androgenic: Central nervous system actions of androgens (e.g., testosterone, dehydroepiandrosterone) or on androgen receptors, refers to “masculinizing” hormones.
Estrogenic: Central nervous system actions of estrogens or on estrogen receptors.
